# Volatile, Lipidomic and Transcriptomic Profiles of Longissimus Muscle from Yunong Black and Landrace Pigs: An Exploratory Comparison

**DOI:** 10.3390/foods15213973

**Published:** 2026-11-05

**Authors:** Tengfei Wang, Xiaotong Wang, Kaiyuan Zhang, Chang Wang, Tong Yu, Feng Yang, Xiuling Li, Jiuhong Nan, Xuelei Han, Xinjian Li

**Affiliations:** 1 College of Animal Science and Technology, Henan Agricultural University, Zhengzhou 450046, China; tfwang1006@163.com (T.W.);; 2 College of Animal Science and Technology, Henan University of Animal Husbandry and Economy, Zhengzhou 450046, China; 3 The Second Affiliated Hospital & Liangzhu Laboratory, Zhejiang University School of Medicine, Hangzhou 311121, China; 4 Animal Husbandry and Veterinary Research Institute, Hainan Academy of Agricultural Sciences, Haikou 570100, China

**Keywords:** pork aroma, volatile compounds, lipid metabolism, flavor-directed breeding

## Abstract

The molecular basis underlying the characteristics of the newly bred Yunong Black (YN) pork remains unclear. Here, we integrated flavoromics, lipidomics, and transcriptomics to characterize breed-associated differences in pork flavor-related traits between YN and Landrace (L) pigs. Transcriptomic and lipidomic analyses revealed breed-associated differences in the expression of genes related to lipid metabolism and in muscle lipid composition. Although exploratory integrative correlation analyses identified pairwise correlations among selected genes, lipid species, and volatile compounds, none of the volatile–gene or gene–lipid associations remained significant after adjustment for breed. These findings highlight the importance of accounting for breed effects when interpreting multi-omics correlations and provide fundamental data on the molecular features associated with breed-related variation in pork flavor. These findings provide a basis for further investigations into the molecular features associated with variation in pork quality and flavor-related traits.

## 1. Introduction

Pork is one of the most widely consumed animal proteins globally, and its flavor is a key determinant of consumer preference and commercial value. However, modern breeding programs that prioritize production traits, such as rapid growth and feed efficiency, have inadvertently reduced intramuscular fat (IMF) content and limited the accumulation of key flavor precursors, including free amino acids, phospholipids, and thiamine, potentially compromising the flavor characteristics of commercial lean pig breeds [1]. In contrast, Chinese indigenous pig breeds exhibit distinct genetic backgrounds and metabolic characteristics that contribute to distinctive meat-flavor characteristics, offering a valuable reservoir of alleles for meat-quality improvement [2,3]. Recent efforts to incorporate indigenous germplasm into commercial lines have demonstrated the potential to combine high growth performance with desirable flavor characteristics, thereby supporting breeding strategies that balance meat quality and production efficiency [4,5]. Meat flavor is a complex trait influenced by volatile organic compounds (VOCs), non-volatile taste components, and textural properties [6]. Among these factors, lipids play a central role, particularly phospholipids, which are rich in polyunsaturated fatty acids and can undergo oxidative degradation to generate volatile compounds, including aldehydes and ketones, thereby contributing to the characteristic aroma of pork and influencing its oxidative stability [7,8]. Despite the recognized importance of lipids in flavor formation, the molecular networks linking lipid metabolism, gene expression, and volatile compound profiles in pig muscle remain poorly understood.

Recent advances in multi-omics technologies offer unprecedented opportunities to dissect the molecular architecture of meat flavor [9,10]. Transcriptomic analysis provides insights into gene-expression patterns associated with muscle development, lipid metabolism, and oxidative processes, with candidate genes such as *FABP4* and *SCD* implicated in lipid metabolism [11,12]. Lipidomics, typically performed using UHPLC–MS/MS, enables comprehensive profiling of lipid classes and molecular species, including triglycerides, phospholipids, and free fatty acids, some of which may contribute to the formation of flavor-related compounds [13,14]. Flavoromics characterizes the chemical composition of flavor-related compounds, including VOCs, that contribute to meat aroma. Integrating these omics datasets makes it possible to map the co-variation among gene-expression patterns, lipid species, and VOC profiles, facilitating the generation of hypotheses about candidate genes and metabolic pathways associated with meat flavor variation [15,16]. However, studies integrating transcriptomics, lipidomics, and flavoromics to investigate breed-associated differences in pork flavor remain limited.

The YN pig is a recently developed composite breed derived from a multi-breed crossbreeding program involving Duroc, Laiwu Black, Nanyang Black, and Erhualian pigs, with the aim of improving growth performance while retaining the favorable IMF content and flavor characteristics of indigenous Chinese pig breeds [17]. Their distinctive genetic background provides an opportunity to investigate the molecular features associated with breed-related differences in pork flavor. In this study, we performed an integrated multi-omics analysis of the longissimus thoracis et lumborum (LTL) muscle from YN and L pigs by integrating transcriptomics, untargeted lipidomics, and flavoromics. Specifically, our objectives were to (i) characterize breed-associated differences in gene expression, lipid composition, and volatile compound profiles; (ii) construct an integrated gene–lipid–flavor co-variation map to explore the molecular relationships underlying differences in pork aroma; and (iii) identify candidate genes and metabolic pathways associated with flavor variation that may inform flavor-oriented breeding strategies. Our findings provide insights into the molecular features associated with breed-related differences in pork flavor and may inform future efforts to improve pork quality.

## 2. Materials and Methods

### 2.1. Ethics Statement

All animal procedures were approved by the Ethics Committee of Henan Agricultural University (Approval No. HNND2026031016).

### 2.2. Animals, Husbandry, Slaughter, and Sample Collection

A total of eight gilts were used in this study, including four Yunong Black gilts (YN, *n* = 4) and four Landrace gilts (L, *n* = 4). To minimize environmental variation between breed groups, all pigs were reared under the same indoor housing and management conditions and received the same complete diet, with unrestricted access to feed and drinking water. The basal diet was formulated according to the Chinese National Standard Nutrient Requirements of Pigs in China (GB/T 39235–2020). The basal diet was provided by Beijing Dabeinong Technology Group Co., Ltd. (Beijing, China) [18]. The detailed ingredients and nutrient composition are presented in Supplementary Table S1. Both breeds were maintained under the same feeding regimen to minimize the potential influence of dietary conditions on meat quality and muscle lipid deposition.

The YN population used in this study originated from the seventh-generation (G7) breeding base population. All pigs were slaughtered at approximately 100 kg body weight. The YN pigs reached this slaughter weight at approximately 175 days of age, whereas the L pigs reached the same body weight at approximately 145 days. Thus, the two breeds were compared at a similar production endpoint rather than at the same chronological age. The eight experimental animals originated from eight different litters. Before slaughter, all pigs were fasted for 12 h with free access to water. Animals were electrically stunned at 1.3 A for 10 s and subsequently exsanguinated by severing the carotid artery. Samples of the LTL muscle were collected from the left side of each carcass at the third to fourth thoracic vertebrae within 30 min postmortem. Samples intended for biochemical and molecular analysis were immediately snap-frozen in liquid nitrogen and stored at −80 °C.

### 2.3. Meat Quality and Intramuscular Fat Measurements

Muscle pH, meat color, and intramuscular fat (IMF) content were determined using standard procedures. Muscle pH was measured using a portable pH meter (Hanna HI98163, Hanna Instruments, Woonsocket, RI, USA) equipped with a glass-tip probe. The meter was calibrated daily using a two-point calibration with standard buffers at pH 4.01 and 7.00, and recalibration was performed when the calibration error exceeded 0.05 pH units. The probe was inserted directly into the collected LTL muscle samples, avoiding visible adipose and connective tissues. Meat color parameters (L*, a*, and b*) were measured using a Minolta CM-508d spectrophotometer (Konica Minolta, Tokyo, Japan) with illuminant A (2854 K), a 10° standard observer angle, and an 8-mm aperture. Before measurement, freshly cut muscle surfaces were allowed to bloom for 30 min at 4 °C. Three readings were obtained from each sample, and the mean value was used for subsequent analyses. IMF content was determined by Soxhlet extraction using freeze-dried muscle samples. Briefly, the freeze-dried samples were weighed and placed in filter paper thimbles, followed by extraction with petroleum ether for 6–8 h. After extraction, the solvent was recovered, and the extraction flasks were dried at 101–105 °C to constant weight. IMF content was determined by Soxhlet extraction using freeze-dried and powdered LTL muscle samples. IMF content was expressed as grams per 100 g of dry matter (g/100 g dry matter).

### 2.4. Flavoromics Analysis

Flavoromics profiling of raw (uncooked) LTL muscle was performed by Biotree Biotechnology Co., Ltd. (Shanghai, China). Samples stored at −80 °C were thawed at 4 °C before volatile metabolite compound extraction. Peak areas were normalized by the service provider to the internal standard added to each vial, and this internal-standard scale is the basis of every breed comparison of individual volatiles reported in this paper.

Sample preparation and headspace solid-phase microextraction (HS-SPME). Muscle samples were cryogenically pulverized in liquid nitrogen for 3 min. A 1 g aliquot of the resulting powder was transferred to a 20 mL headspace vial, and 10 μL of internal standard solution was added. The internal standard was n-hexyl-d_13_ alcohol (CAS No. 204244-84-8; 1 mg/L in 50% aqueous ethanol; C/D/N Isotopes, QC, Canada). No salt was added, and the vials were not agitated. Volatile compounds were extracted using a divinylbenzene/carboxen/polydimethylsiloxane (DVB/CAR/PDMS) fiber (50/30 μm film thickness, 1 cm length; Supelco 57329-U, Bellefonte, PA, USA). The fiber was conditioned at 270 °C for 10 min before extraction and reconditioned after injection. The vials were equilibrated at 80 °C for 10 min, after which the fiber was exposed to the headspace at 80 °C for 40 min. The extracted analytes were desorbed in the GC inlet at 250 °C for 5 min. Linear retention indices (LRIs) were calibrated using a C7–C30 n-alkane series (10 μL; 1 mg/L in n-hexane) analyzed under the same chromatographic conditions.

GC × GC–TOF MS conditions. Volatile compounds were analyzed on a LECO Pegasus BT 4D comprehensive two-dimensional gas chromatography–time-of-flight mass spectrometry (GC × GC–TOF MS) system (LECO, St. Joseph, MI, USA) consisting of an Agilent 8890A gas chromatograph (Agilent Technologies, Palo Alto, CA, USA) fitted with a split/splitless injector and a dual-stage cryogenic modulator and coupled to a high-resolution time-of-flight mass spectrometer. The first-dimension column was a DB-Heavy Wax column (30 m × 250 μm i.d., 0.5 μm film thickness; Agilent, Santa Clara, CA, USA), and the second-dimension column was an Rxi-5Sil MS column (2.0 m × 150 μm i.d., 0.15 μm film thickness; Restek, Bellefonte, PA, USA). Helium (>99.999%) was used as carrier gas at a constant flow of 1.0 mL/min. The primary oven was held at 50 °C for 2 min, raised to 220 °C at 4 °C/min, and held for 13 min; the secondary oven was maintained 5 °C above the primary oven and the modulator 15 °C above the secondary oven, with a modulation period of 5.0 s. The injector temperature was 250 °C. The transfer line and ion source were both held at 250 °C; spectra were acquired at 200 spectra/s in electron-impact mode at 70 eV over *m*/*z* 35–550, and the detector voltage was 2020 V.

Compound identification and classification. Chromatographic peaks were integrated using ChromaTOF software (version 4.3x). Volatile compounds were annotated by matching their mass spectra against the NIST 2020 library and comparing their experimental LRIs, calculated from the C7–C30 n-alkane series, with corresponding library values. For this study, compounds were assigned to two identification categories: RI-supported, compounds with an experimental LRI within 20 units of the corresponding library value; and MS-only, compounds annotated by mass spectral matching when a corresponding library LRI was unavailable. The experimental and library LRIs, the differences between them, and the assigned identification categories are reported in Supplementary Table S4. These categories are study-specific and are deliberately left unnumbered. Under the Metabolomics Standards Initiative scheme, a level 1 identification requires comparison with an authentic chemical standard analyzed under the same conditions; no authentic chemical standard was analyzed in this study, so no compound reported here reaches MSI level 1, and both categories used here lie below it. The library LRIs used in the comparison were taken for a polar (wax-type) stationary phase, consistent with the DB-Heavy Wax first-dimension column. The annotated compounds were classified into hierarchical chemical categories using the PubChem database and ClassyFire (version 0.3.8). The numbers of detected compounds and their relative abundances were summarized by chemical category to characterize differences in volatile composition between the two breeds. Odor descriptors for the identified compounds were obtained from the FlavorDB database.

Relative odor activity value (ROAV) estimation and statistical analysis. ROAV was calculated for those of the 221 volatile compounds that were carried into the statistical analysis and for which a published odor threshold was available. Odor thresholds were taken from published compilations of sensory detection thresholds, in which the values have been determined predominantly in water, air, or other simple model systems rather than in meat; the matrix of determination is not recorded for the individual entries of the table supplied by the service provider. The threshold value and the odor descriptor used for each compound are listed in Supplementary Table S6. For each sample, the abundance of a compound was expressed relative to the total signal of those 221 compounds in the same sample. The relative abundance of each compound was divided by its corresponding odor threshold to obtain a threshold-weighted index. These indices were then normalized relative to the compound with the highest threshold-weighted index, using a common reference across both breeds. Because absolute volatile concentrations were not measured, these ROAVs should be interpreted as exploratory, relative indices rather than quantitative measures of odor activity. ROAV of 1 is an internal normalization reference, not a sensory threshold. Differences in the relative abundances of individual volatile compounds between YN and Landrace pigs were assessed using Student’s *t*-test. Differential volatile compounds were defined as those meeting both criteria: an absolute log_2_ fold change (|log_2_FC|) ≥ 1 and a Benjamini–Hochberg false discovery rate (FDR) < 0.05. FDR values were calculated from statistical comparisons of log_2_-transformed normalized abundances. Normalization underlying each reported result. Two relative scales are used for the volatile data, and each reported result is tied to one of them: (i) Internal-Standard Normalization: Peak areas were divided by the peak area of the n-hexyl-d13 alcohol added to each vial before extraction. It is a ratio to a single added standard rather than a calibration against external standards or compound-specific response factors, so it remains a relative scale and does not yield absolute concentrations. (ii) Total Annotated Volatile Signal: For the ROAV calculation only, each peak area was expressed as a percentage of the total annotated volatile signal of the same sample. The ROAV values are used only to rank compounds within the data set, and no result is transferred from one scale to the other.

### 2.5. Lipidomics Analysis

Lipidomic profiling of the LTL muscle was performed using a UHPLC–MS/MS platform by Biotree Biotechnology Co., Ltd. (Shanghai, China). Approximately 50 mg of frozen LTL tissue was weighed into a 2 mL microcentrifuge tube containing a 6 mm grinding bead. Subsequently, 280 μL of methanol/water (2:5, *v*/*v*) and 400 μL of methyl tert-butyl ether (MTBE) were added to each sample. The samples were homogenized at −10 °C using a high-throughput tissue grinder at 50 Hz for 6 min, followed by ultrasonication at 40 kHz for 30 min at 5 °C. The homogenates were then incubated at −20 °C for 30 min and centrifuged at 13,000× *g* for 15 min at 4 °C. After centrifugation, 350 μL of the upper organic phase was carefully transferred to a clean tube. The collected organic phase was evaporated to dryness under a gentle stream of nitrogen. The dried lipid extracts were reconstituted in 200 μL of isopropanol, followed by filtration through a 0.22 μm PTFE membrane before instrumental analysis.

Lipidomic analysis was performed on a Vanquish UHPLC system (Thermo Fisher Scientific, Waltham, MA, USA) coupled to a Q Exactive mass spectrometer (Thermo Fisher Scientific, Waltham, MA, USA). The conditions below are those recorded in the provider’s acquisition report. Chromatographic separation was carried out on an ACQUITY UPLC BEH C18 column (2.1 × 100 mm, 1.7 μm; Waters, Milford, MA, USA) maintained at 50 °C, with the autosampler held at 8 °C. The mobile phases were acetonitrile–water (60:40, *v*/*v*) containing 0.1% formic acid and 10 mM ammonium formate (A) and isopropanol–acetonitrile (90:10, *v*/*v*) containing 0.1% formic acid and 10 mM ammonium formate (B), delivered at 0.25 mL/min; the injection volume was 2 μL. The elution program was 0–5 min, 70–57% A; 5–5.1 min, 57–50% A; 5.1–14 min, 50–30% A; 14–14.1 min, 30% A; 14.1–21 min, 30–1% A; 21–24 min, 1% A; 24–24.1 min, 1–70% A; and 24.1–28 min, 70% A. Mass spectrometric detection used electrospray ionization in both positive and negative modes, with spray voltages of 3.5 kV and 2.5 kV, sheath gas at 30 arbitrary units, auxiliary gas at 10 arbitrary units, and a capillary temperature of 325 °C. Full-scan spectra were acquired over *m*/*z* 150–2000 at a resolution of 35,000, and data-dependent MS/MS spectra were acquired by higher-energy collisional dissociation at a normalized collision energy of 30 eV with dynamic exclusion. Lipid species were identified using LipidSearch software (version 4.2.28) based on precursor and fragment ion information, with a retention-time tolerance of 0.25 min and an m-score threshold of 3. The resulting lipid matrix contained lipid identities, retention times, *m*/*z* values, and peak intensities. To account for differences in overall signal intensity among samples, peak intensities were normalized to the total peak area (sum-peak normalization), as reported in Supplementary Table S7 and Supplementary Figure S2. The normalized values were interpreted as relative lipid abundances rather than absolute lipid concentrations. According to the service provider’s standard operating procedure, pooled quality-control (QC) samples are prepared by combining aliquots of the extracted study samples, and QC injections are scheduled only for analytical cohorts comprising more than 20 samples. As this study included eight samples, no pooled-QC injections were performed. Consequently, injection-level repeatability filtering based on relative standard deviation (RSD) and QC-based signal drift correction were not applied. Extraction blanks (process blanks) were not included, and no documentation of injection-order randomization was available. No class-specific isotope-labeled internal standards or absolute-concentration calibration was used. The two within-class metrics were calculated from the annotated chain compositions of individual lipid features. A feature was classified as containing an 18:1 or 20:1 fatty acyl chain only when its annotation resolved the individual chains. Features annotated only at the species (sum-composition) level were excluded from the 18:1/20:1 group because their individual chain compositions could not be determined. No chain composition was inferred from the total carbon number and double-bond count. The signals of these unresolved features were retained in the total signal for the corresponding lipid class used as the denominator. For sphingolipids, the sphingoid base was distinguished from the fatty acyl chain when interpreting chain composition.

### 2.6. RNA Extraction and Transcriptome Analysis

Total RNA was extracted from LTL tissues using RNAiso Plus (TaKaRa, Beijing, China). Raw sequencing reads were subjected to quality control using fastp (v0.23.2). The resulting clean reads were aligned to the porcine reference genome (Sscrofa11.1; GCA_000003025.6) using HISAT2 (v2.2.1), with gene annotations obtained from Ensembl release 105. Gene-level read counts were generated using featureCounts (v2.0.3). Differentially expressed genes (DEGs) between YN and L pigs were identified using the DESeq2 R package (v1.36.0), with thresholds of |log_2_ fold change| ≥ 1 and Benjamini–Hochberg-adjusted *p* < 0.05. Log_2_ fold changes represent the unshrunken maximum-likelihood estimates obtained from DESeq2. Raw and Benjamini–Hochberg-adjusted *p* values for all genes tested are provided in Supplementary Table S3. Functional enrichment analyses were conducted using the clusterProfiler R package (v4.2.1) to identify enriched Gene Ontology (GO) terms and Kyoto Encyclopedia of Genes and Genomes (KEGG) pathways. The enrichment was run on the DEG set defined above, separately for the transcripts higher in YN, those higher in L, and the two directions combined, with the 16,161 genes tested by DESeq2 as the background set. GO was analyzed on biological process terms annotated to 10–1000 background genes and KEGG on pathways annotated to at least five. *p* values were obtained with a one-sided hypergeometric test and corrected by the Benjamini–Hochberg procedure across all terms tested in the same direction. The analysis was repeated independently from the raw annotations with an explicit hypergeometric test and returned the same terms. Quantitative real-time PCR (qRT-PCR) was performed to validate the expression patterns of selected candidate genes identified by RNA-seq. Gene-specific primers were used for target gene amplification, including *TMEM238* (F: TGCTGACCGGCGTGTTC, R: ATGTTGCCGGTGTACCAGAG, 127 bp), *FASN* (F: GGTCACCTACGAGGCCATTG, R: TGATGCTGGGCCCCTTGAA, 215 bp), *ZNF865* (F: CTGAGCGTTTTAGCTGTGCC, R: GATGCAGGAGCAGGTCGTAG, 150 bp), *ETV4* (F: GTACCAGACAGCGATGAGCA, R: GGGGTCATAGGCACTGGAAT, 189 bp), and *GAPDH* (F: ATCCATGACCACTTCGGCATC, R: AAGCAGGGATGATGTTCTGGG, 139 bp) served as the reference gene for normalization. Relative gene expression levels were compared between YN and L pigs.

### 2.7. Correlation Analysis

Integrative correlation analysis was performed to investigate the relationships among differentially abundant volatile organic compounds (VOCs), differentially expressed genes (DEGs), and differentially abundant lipids (DELs). The analysis included the 10 differential VOCs, 40 DEGs selected based on differential expression significance and annotation, and 176 differential lipid features. These feature sets yielded 400 VOC–gene, 1760 VOC–lipid, and 7040 gene–lipid pairwise tests. The DEGs were selected from transcripts meeting the criteria of adjusted *p* < 0.05 and |log_2_ fold change| ≥ 1. Because all input features were preselected based on breed-associated differences, the resulting correlations may be influenced by feature selection and shared breed effects. Partial-correlation analyses were subsequently performed to account for breed effects, and the resulting *p* values were adjusted for multiple testing using the false discovery rate (FDR) procedure. Three pairwise analyses were conducted: VOC–lipid, gene–lipid, and gene–VOC correlations. Pearson’s correlation coefficient was calculated using the OmicShare platform (https://www.omicshare.com/, accessed on 5 May 2026). For visualization, the top 10 feature pairs in each comparison were selected based on the absolute correlation coefficient. Benjamini–Hochberg false discovery rate (FDR) correction was applied separately to the three sets of pairwise tests. A tripartite gene–lipid–VOC co-variation map was constructed by integrating the pairwise correlations, using (|r| ≥ 0.8) as the correlation-strength threshold. Correlations were calculated across all eight samples (four per breed; *n* = 8, df = 6 for the unadjusted Pearson correlations). At this sample size, (|r| ≥ 0.8) corresponds approximately to a nominal two-sided (*p* < 0.02) before multiple-testing correction. To assess the potential influence of breed differences on the observed associations, partial correlations adjusted for breed were additionally calculated as a robustness check. Given the limited sample size and the inclusion of two genetically distinct breeds, the resulting network was interpreted as an exploratory representation of cross-sample covariation among breed-differential features rather than evidence of direct regulatory or causal relationships. Betweenness centrality was calculated and reported descriptively, without using it as a criterion for identifying candidate regulatory genes. The network was visualized as a tripartite node–link map, with hierarchical clustering applied to the correlation patterns.

### 2.8. Statistical Analysis

Statistical analysis was performed using SPSS version 26.0 (IBM Corp., Road Armonk, NY, USA). Data are presented as mean ± standard deviation (SD). Differences between the two groups were assessed using Student’s *t*-test. Differentially abundant metabolites and VOCs were identified based on Benjamini–Hochberg-adjusted *p* values. For VOCs, an additional absolute log_2_ fold-change threshold of ≥1 was applied. Statistical significance was defined as * *p* < 0.05, ** *p* < 0.01, and *** *p* < 0.001, respectively.

## 3. Results

### 3.1. Meat Quality Traits of YN and L Pig Muscle Samples

Meat quality is a critical determinant of consumer acceptance. In this study, meat quality traits were evaluated in the LTL muscle of YN and L pigs. The measured traits, including pH at 45 min and 24 h postmortem, meat color parameters (L*, a*, and b*), and IMF content, are summarized in Table 1. Significant differences between the two breeds were observed in most of the measured traits (*p* < 0.05). Specifically, YN pigs exhibited significantly higher pH values at both 45 min and 24 h postmortem, higher a* values, and greater IMF content, but significantly lower L* values than L pigs (*p* < 0.05).

### 3.2. Breed-Specific Volatile Profiles Distinguish YN and L Pork

**Figure 1:**
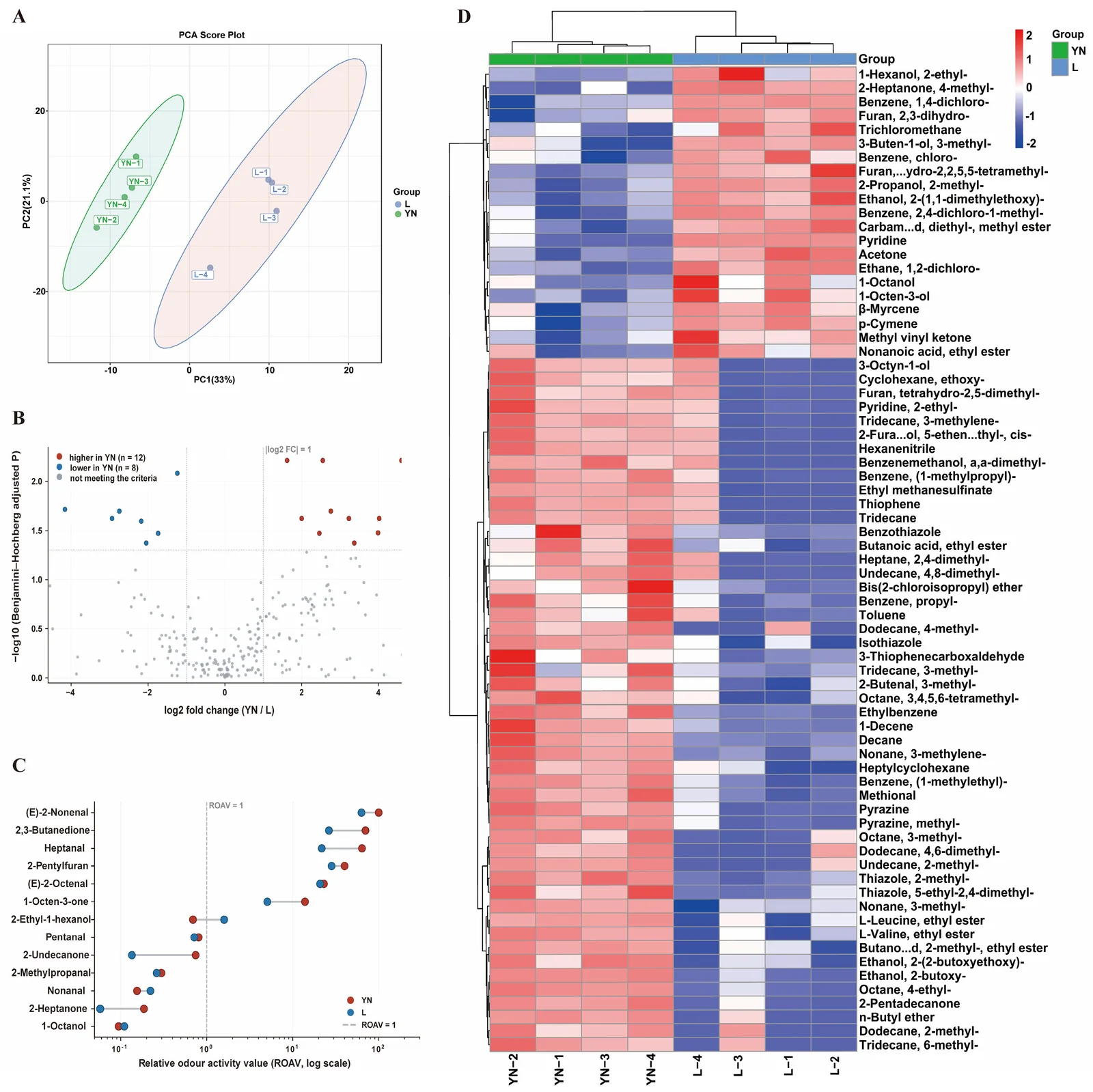
Volatile profiles of YN and L pork (*n* = 4 per breed): (**A**) Principal component analysis (PCA) score plot of volatile organic compound (VOC) profiles. Each point represents a sample, with different colors denoting different breeds; the ellipse shows the 95% confidence interval. (**B**) Volcano plot of differentially abundant VOCs between YN and L pork. The x-axis represents log_2_ fold change (log_2_ FC), and the y-axis represents log_10_ of the Benjamini–Hochberg-adjusted *p* value. Red and blue points indicate VOCs with significantly higher and lower abundances in YN pork, respectively, whereas gray points indicate VOCs that did not meet the differential abundance criteria. (**C**) Relative odor activity values ROAVs of the retained VOCs with published odor threshold values (62 of the 221 compounds retained after the contaminant screen). ROAVs for YN (red) and L (blue) are displayed on a logarithmic scale. The dashed line indicates ROAV = 1, used as a reference threshold for potentially important odor-active compounds. (**D**) Hierarchically clustered heatmap of VOC abundance profiles. Rows represent individual biological samples (YN_1–4 and L_1–4), and columns represent individual VOCs annotated by chemical class. Color intensity indicates z-score-normalized relative abundance (red, higher abundance; blue, lower abundance). The left dendrogram represents sample clustering, and the top dendrogram represents VOC clustering.

Flavoromics analysis revealed distinct volatile profiles between YN and L pigs. Principal component analysis (PCA) showed separation between the two breeds, with PC1 and PC2 explaining 33.0% and 21.1% of the total variance, respectively (Figure 1A). Differential analysis identified altered volatile compounds (Figure 1B). Ranking the annotated volatiles by relative odor activity value (ROAV) placed seven of the 90 threshold-annotated compounds above the internal reference in at least one breed—(E)-2-nonenal, 2,3-butanedione, heptanal, 2-pentylfuran, (E)-2-octenal, 1-octen-3-one, and 2-ethyl-1-hexanol—and six of these seven were shared by both breeds, so the top-ranking set was largely common to YN and L pork (Figure 1C; Supplementary Table S6). Analysis of odor descriptors indicated that green, fatty, nutty, and floral notes accounted for larger proportions in L pork, whereas buttery, citrus, rancid, and mushroom notes were more prominent in YN pork (Supplementary Figure S1, Supplementary Table S6). Hierarchical clustering further demonstrated breed-associated patterns in volatile compound abundance, with several aldehydes, ketones, and sulfur-containing compounds showing higher abundances in YN pork (Figure 1D). Collectively, these results show that YN and L pork differ in the composition of the volatile fraction and in the relative contribution of the individual compounds to it.

### 3.3. Breed-Associated Transcriptomic Differences Between YN and L Pigs

**Figure 2:**
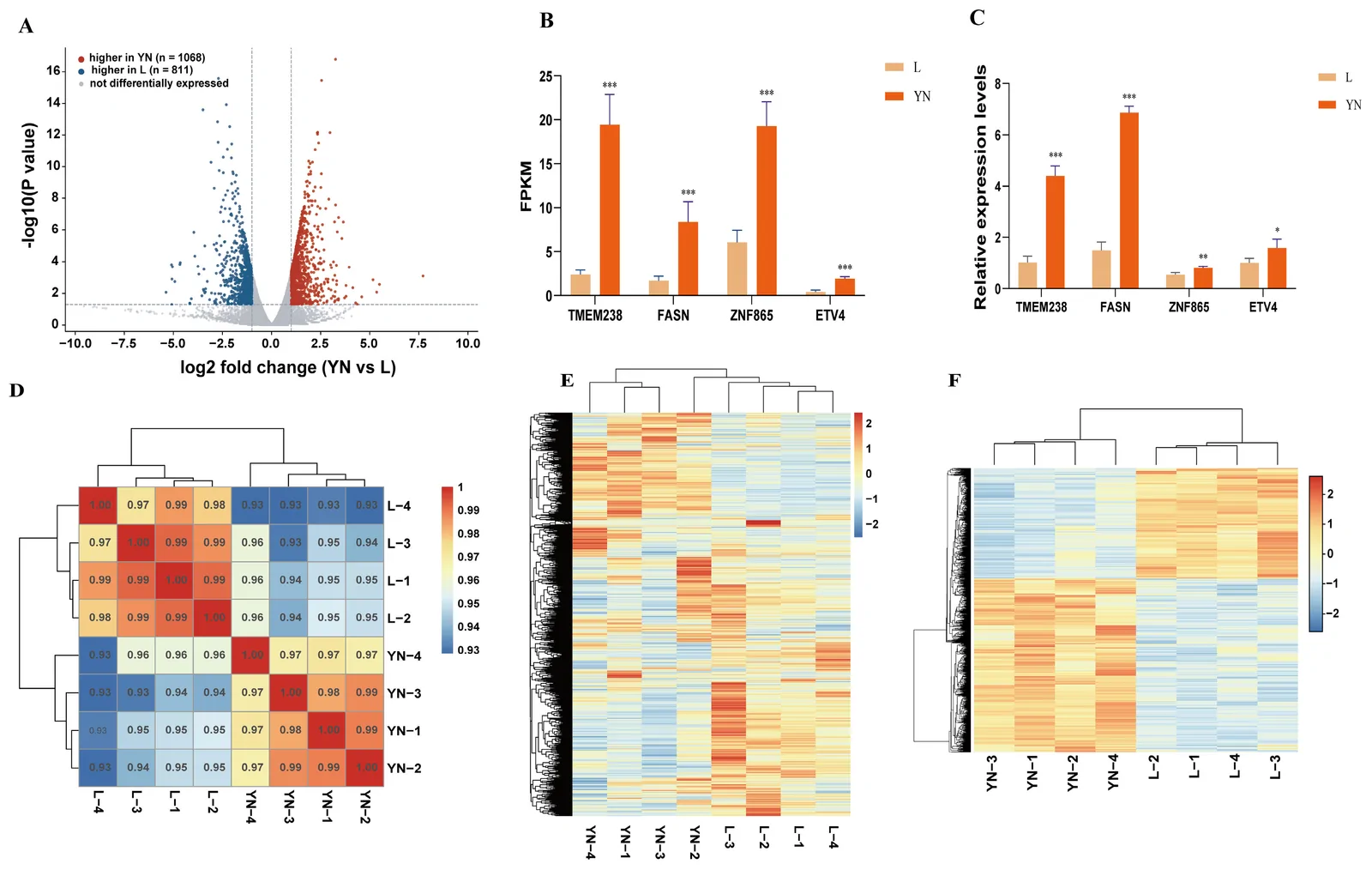
Comparative transcriptome analysis of the LTL muscle of YN and L pigs (*n* = 4 per breed): (**A**) Volcano plot of the 14,755 genes with a defined fold change. The x-axis is the log_2_ fold change for the contrast YN versus L, so that positive values indicate higher expression in YN; the y-axis is −log_10_ of the Benjamini–Hochberg-adjusted *p* value. Red, the 794 genes that are higher in YN and meet the DEG criterion; blue, the 504 genes that are higher in L and meet it; grey, genes that meet neither condition. (**B**) FPKM values from RNA-seq for four candidate genes. Significance levels are indicated as * *p* < 0.05, ** *p* < 0.01, and *** *p* < 0.001. (**C**) Relative expression of the same four genes measured by qRT-PCR, with GAPDH as the reference gene; B and C are independent measurements on the same biological replicates. Significance levels are indicated as * *p* < 0.05, ** *p* < 0.01, and *** *p* < 0.001. (**D**) Pearson’s correlation coefficient between the four biological replicates of the two breeds. (**E**) Heatmap of the expression of all genes detected in the muscle samples. Rows and columns were clustered by average linkage. (**F**) Heatmap of the 1298 transcripts that meet the DEG criterion, ordered by hierarchical clustering; color is the row-scaled z-score of log_2_ (FPKM + 1).

RNA sequencing was performed to characterize transcriptomic differences between YN and L pigs. Following quality control and genome alignment, high-quality transcriptomic profiles were obtained from LTL muscle samples. Differential expression analysis identified 1298 DEGs between the two breeds, of which 794 were more highly expressed in YN pigs and 504 more highly expressed in L pigs (Figure 2A). Among these, four selected differentially expressed genes, including *TMEM238*, *FASN*, *ZNF865*, and *ETV4*, showed higher expression in YN than in L (Figure 2B). The expression patterns of these genes were further examined by qRT-PCR, which confirmed significantly higher expression in YN pigs for all four genes, consistent with the RNA-seq results (Figure 2C). Sample correlation analysis showed high within-group correlations (r = 0.97–0.99), whereas correlations between YN and L samples were comparatively lower (r = 0.93–0.97) (Figure 2D). Consistently, hierarchical clustering and heatmap analyses revealed breed-associated gene expression patterns, with samples clustering according to breed (Figure 2E,F). To further characterize the transcriptional differences, the 20 DEGs with the smallest adjusted *p* values among those higher in YN and the 20 with the smallest adjusted *p* values among those higher in L are listed in Table 2.

**Figure 3:**
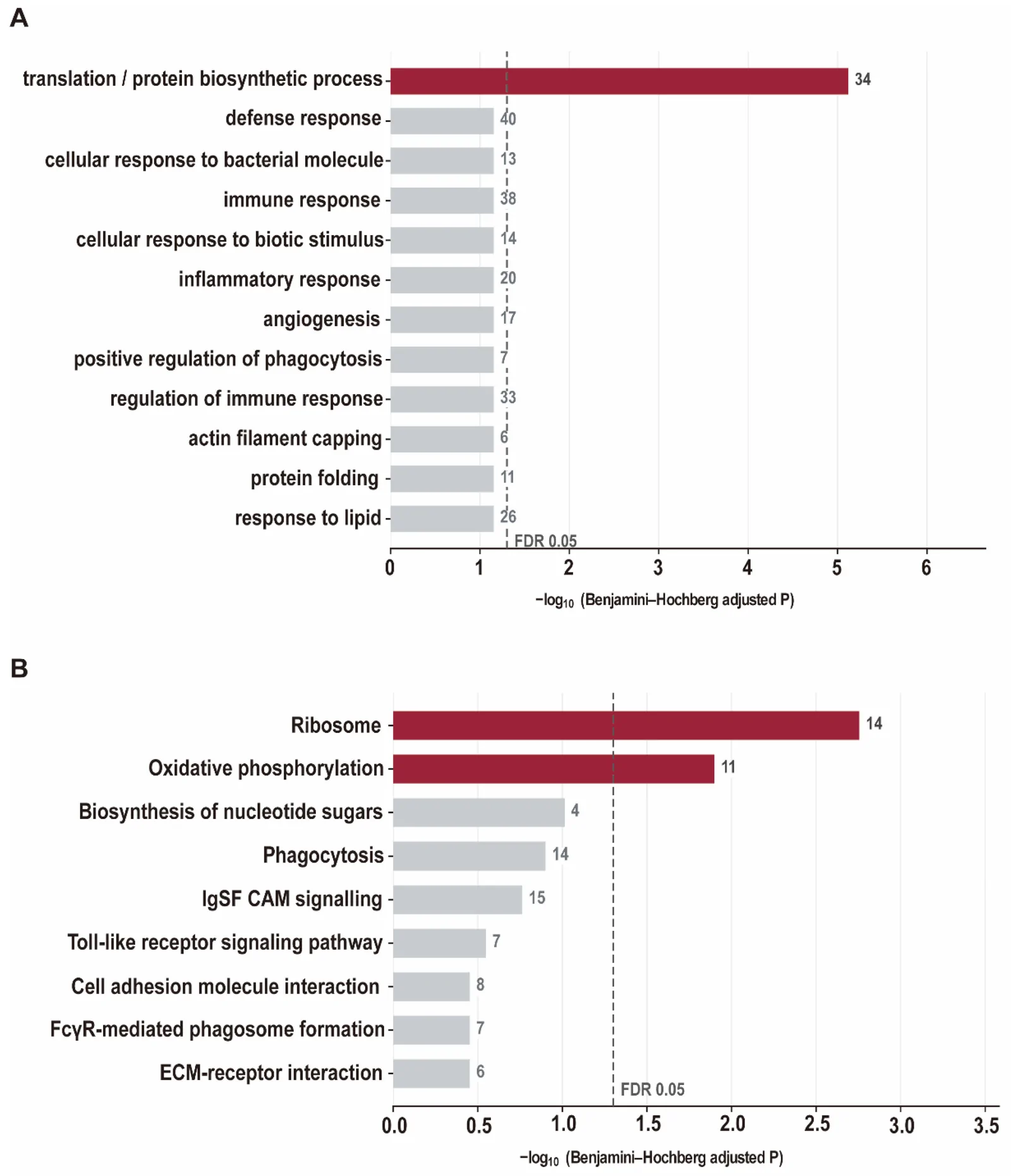
Functional enrichment of the differentially expressed transcripts: (**A**) GO enrichment analysis between YN and L. (**B**) KEGG enrichment analysis of DEGs between YN and L.

To characterize the biological processes and pathways associated with the transcriptomic differences, GO and KEGG enrichment analyses were performed on the 1298 DEGs, separately for the transcripts higher in YN, those higher in L, and the two directions combined. Among the transcripts higher in L (*n* = 504), GO analysis tested 3060 biological process terms, and two passed at adjusted *p* < 0.05; both describe translation and contain the same 34 transcripts (adjusted *p* = 7.6 × 10^−6^), so they are shown as a single term in Figure 3A. KEGG analysis tested 175 pathways, of which two passed: ribosome (14 transcripts, adjusted *p* = 1.8 × 10^−3^) and oxidative phosphorylation (11 transcripts, adjusted *p* = 1.3 × 10^−2^; Figure 3B).

### 3.4. Distinct Lipidomic Profiles Between YN and L Pigs

**Figure 4:**
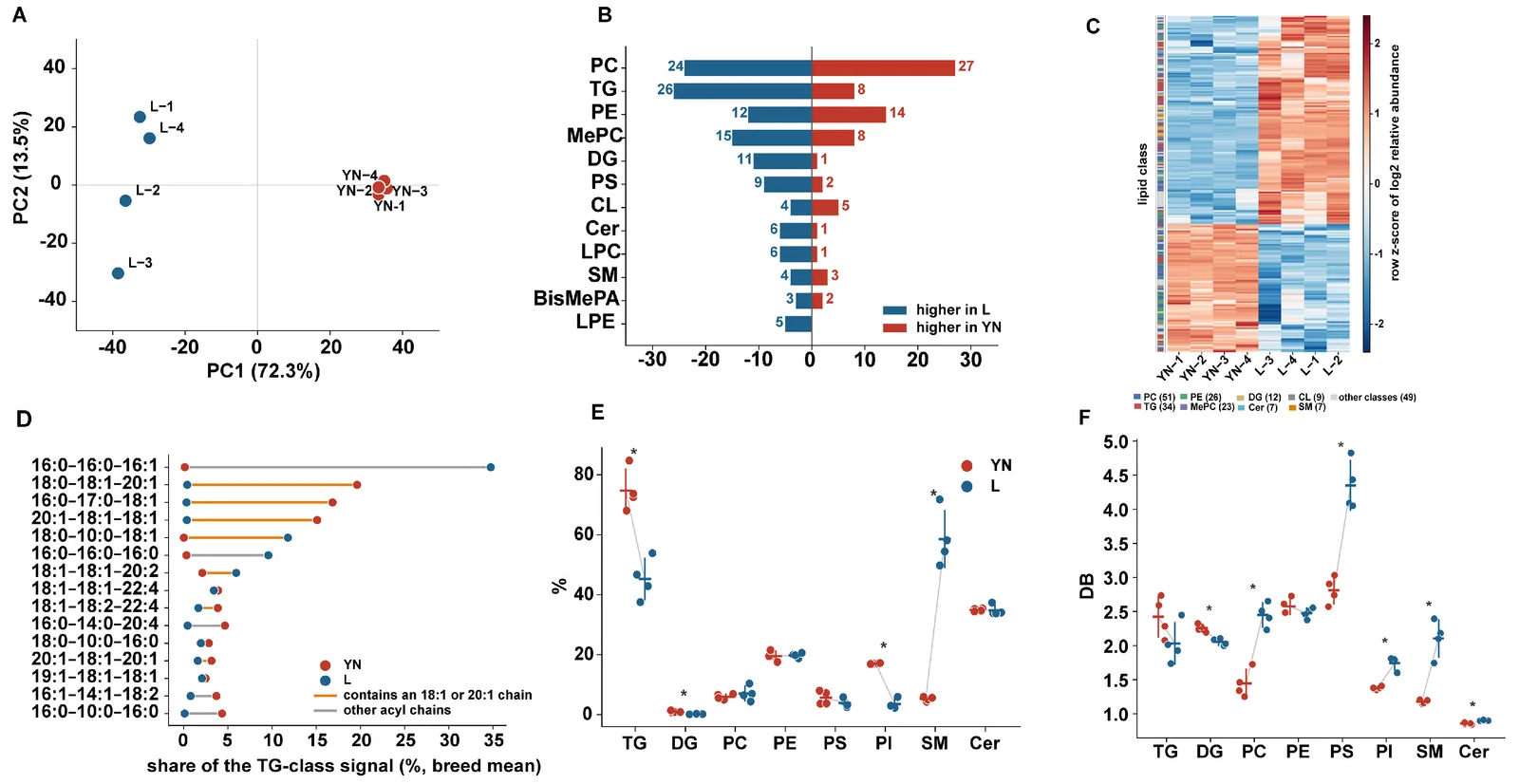
Lipid composition of the LTL muscle of YN and L pigs (*n* = 4 per breed): (**A**) Principal component analysis (PCA) of the 380 curated lipid features without missing values, quantified across all eight samples using log_2_-transformed relative abundances. PC1 and PC2 explain 72.5% and 13.6% of the total variance, respectively. (**B**) Number of differentially abundant lipid features in each lipid class, identified using Student’s *t*-test. (**C**) Hierarchical clustering of the 176 curated differential features using Pearson correlation and average linkage. Rows represent samples, and log_2_-transformed relative abundances were z-score-normalized for visualization. The color strip on the left indicates lipid class. (**D**) Within the TG class, the proportion of the total TG signal attributable to each of the 15 most abundant TG species, ranked by their mean abundance across the two breeds. Lines connect the mean values for each breed, and orange markers identify species containing an 18:1 or 20:1 fatty acyl chain. (**E**) Proportion of the total signal within each lipid class attributable to species containing an 18:1 or 20:1 fatty acyl chain. (**F**) Signal-weighted mean number of double bonds per lipid molecule within each lipid class. In (**E**,**F**), symbols are the individual animals (*n* = 4 per breed), and the two breeds are compared within each class by Welch’s *t*-test; * *p* < 0.05. Lipid classes are abbreviated as follows: TG, triacylglycerol; DG, diacylglycerol; PC, phosphatidylcholine; PE, phosphatidylethanolamine; PS, phosphatidylserine; PI, phosphatidylinositol; SM, sphingomyelin; and Cer, ceramide.

Lipidomics was performed to characterize lipid composition between YN and L pigs. The identified lipid species and their corresponding retention times (RT), mass-to-charge ratios (*m*/*z*), and other analytical information are provided in Supplementary Table S2. PCA of the 380 curated features without missing values showed clear separation between YN and L pigs, with PC1 and PC2 explaining 72.5% and 13.6% of the total variance, respectively (Figure 4A). Differential abundance analysis of the 392 curated features identified 176 lipid species with significant breed-associated differences after Benjamini–Hochberg correction (adjusted *p* < 0.05), of which 61 had higher and 115 had lower relative abundances in YN pigs (Figure 4B). The direction of differential abundance varied among lipid classes, although lower relative abundances in YN pigs predominated among TG, DG, PS, LPC, and LPE species. Specifically, 26 of 34 TG species, 11 of 12 DG species, 7 of 9 PS species, 6 of 7 LPC species, and all 5 LPE species had lower relative abundances in YN pigs. Hierarchical clustering further revealed distinct breed-associated abundance patterns among the differential lipid species, particularly within the TG, PC, PE, and sphingolipid classes (Figure 4C). Differences between breeds were also observed in the fatty acyl-chain composition of individual lipid classes. The proportion of the class-specific signal attributable to species containing an 18:1 or 20:1 fatty acyl chain was higher in YN pigs than in L pigs for TG (74.8 ± 7.1% vs. 45.3 ± 6.9%, *p* = 0.001), PE (25.2 ± 3.2% vs. 17.6 ± 2.0%, *p* = 0.010), and PI (17.1 ± 0.1% vs. 3.5 ± 1.7%, *p* < 0.001). In contrast, this proportion was lower in YN pigs for SM (5.3 ± 0.7% vs. 58.5 ± 9.5%, *p* = 0.001; Figure 4D,E). The signal-weighted mean number of double bonds per lipid molecule also differed between breeds. Lower values were observed in YN pigs for PC (1.81 ± 0.26 vs. 3.03 ± 0.28, *p* < 0.001), PS (2.81 ± 0.20 vs. 4.35 ± 0.36, *p* < 0.001), PI (1.38 ± 0.02 vs. 1.74 ± 0.10, *p* = 0.004), and SM (1.18 ± 0.03 vs. 2.10 ± 0.27, *p* = 0.006; Figure 4F). Collectively, these results demonstrate breed-associated differences in the relative abundances of individual lipid species, the distribution of fatty acyl-chain compositions across lipid classes, and the signal-weighted degree of unsaturation in selected lipid classes.

### 3.5. Exploratory Integration of Breed-Differential Transcripts, Lipids, and Volatiles

**Figure 5:**
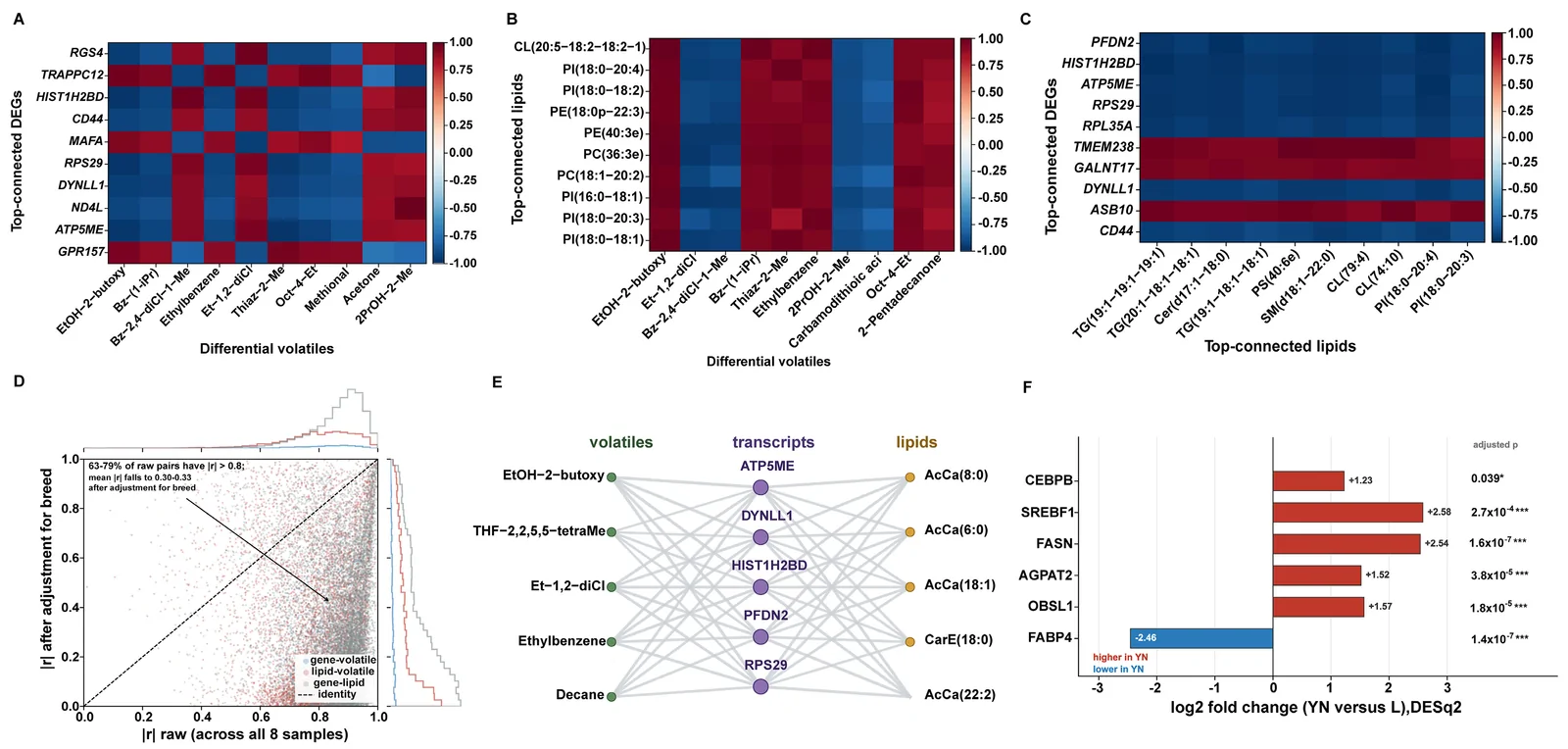
Co-variation of breed-differential transcripts, lipids, and volatiles in YN and L pigs, and differential expression of lipid-metabolism genes: (**A**–**C**) Correlation heatmaps showing the ten most connected features in each pairwise omics layer: (**A**) transcripts versus volatile compounds, (**B**) lipid species versus volatile compounds, and (**C**) lipid species versus transcripts. Colors represent Pearson’s correlation coefficient (r; red, positive correlation; blue, negative correlation). Rows and columns are ordered by hierarchical clustering. (**D**) Comparison of the absolute correlation coefficient (|r|) calculated before (x-axis) and after (y-axis) adjustment for breed using partial correlation analysis. (**E**) Tripartite co-variation network comprising volatile compounds (**left**), the transcripts that were among the ten most connected in both gene-containing layers (center: RGS4, HIST1H2BD, CD44, RPS29, DYNLL1, ND4L, and ATP5ME), and lipid species (**right**). The tripartite map is an exploratory, hypothesis-generating representation based on 4 animals per breed, and its edges are simple pairwise correlations (|r| ≥ 0.8), not statistically validated associations. (**F**) RNA-seq-based log_2_ fold changes (YN/L) for selected candidate genes. Statistical significance was defined as * *p* < 0.05 and *** *p* < 0.001, respectively.

To explore associations among gene expression, lipid composition, and flavor-related volatile compounds, transcriptomic, lipidomic, and flavoromic data from all eight animals were integrated using Pearson correlation analysis. In the volatile–transcript layer, the two compounds that dominated connectivity before the screen, 2-butoxyethanol and isopropyl benzene, were among those excluded; of the ten compounds that remained, the most connected were 2-methylthiazole (|r| ≥ 0.8 against 38 of the 40 transcripts), methional, and acetone. The ten transcripts with the highest connectivity were *RGS4*, *TRAPPC12*, *CD44*, *HIST1H2BD*, *MAFA*, *RPS29*, *PFDN2*, *DYNLL1*, *ATP5ME*, and *ND4L* (Figure 5A). Highly connected features were also observed in the lipid–volatile and transcript–lipid association layers (Figure 5B,C). Notably, *ATP5ME*, *DYNLL1*, *HIST1H2BD*, *PFDN2*, and *RPS29* were among the ten most connected transcripts in both gene-containing layers, linking volatile compounds and lipid species within the tripartite co-variation map (Figure 5E). To distinguish network-derived associations from biologically motivated candidate selection, potential mechanistic candidates were prioritized separately based on differential expression and supporting evidence from previous studies (Figure 5F). These findings provide a basis for generating hypotheses about molecular processes potentially related to breed-associated differences in pork quality and flavor.

## 4. Discussion

Pork flavor is an important component of meat quality and contributes to consumer acceptance and market value [19]. Duroc, Landrace, and Yorkshire are widely used in commercial pig production, and Duroc × (Landrace × Yorkshire) (DLY) is a common three-way crossbreeding system designed to combine favorable growth performance, feed efficiency, and carcass traits [20]. However, meat quality can vary substantially among commercial genotypes, and defects such as pale, soft, exudative (PSE) and dark, firm, dry (DFD) meat can compromise product quality [21,22]. Chinese indigenous pig breeds have been reported to exhibit distinct meat quality and flavor-related characteristics compared with European commercial breeds [23]. In this study, an integrated analysis of flavoromics, transcriptomics, and lipidomics identified breed-associated differences in the volatile profiles, muscle lipid composition, and gene expression of YN and L pigs. These findings provide complementary molecular and metabolic evidence for characterizing the meat quality attributes of YN pigs and generating hypotheses about the biological factors associated with their distinct chemical profiles. Flavoromics analysis revealed distinct volatile profiles between YN and L pork, with the first two principal components explaining 33.0% and 21.1% of the total variance, respectively. Ranking the annotated volatiles by ROAV placed seven compounds above the internal reference in at least one breed, including (E)-2-nonenal, 2,3-butanedione, heptanal, 2-pentylfuran, (E)-2-octenal, 1-octen-3-one, and 2-ethyl-1-hexanol, and six of the seven were shared by both breeds. The distribution of odor descriptors also differed between breeds, with green, fatty, and nutty notes accounting for larger proportions in L pork, whereas buttery, citrus, and rancid notes were more prominent in YN pork. These descriptors provide a chemical-based characterization of the volatile profiles rather than direct evidence of perceived sensory differences, which would require sensory evaluation. Moreover, several of the most strongly differentiated volatile compounds had relative odor activity values (ROAVs) well below 1, suggesting that their individual contributions to overall aroma may be limited under the conditions evaluated. Among the differential volatile compounds, esters such as ethyl acetate and ethyl butanoate, together with aldehydes such as 3-thiophenecarboxaldehyde and methional, showed higher abundances in YN pork. Esters are commonly associated with fruity and other characteristic odor notes, whereas aldehydes can arise from lipid oxidation and amino acid degradation [24,25,26]. These associations provide a chemical context for interpreting the observed volatile differences, although the contribution of individual compounds to the overall aroma depends on their concentrations, odor thresholds, and interactions with other volatiles. Methional, a sulfur-containing Strecker aldehyde associated with boiled-potato and meaty odor notes, showed a marked abundance difference between breeds. However, its ROAV was below 0.01 in both groups, indicating that it was unlikely to make a substantial individual contribution to aroma under the conditions examined. Importantly, all samples were analyzed in the raw, unheated state. The detected compounds therefore characterize the volatile profiles present before cooking and cannot be assumed to represent the aroma generated during thermal processing.

Transcriptomic analysis further revealed breed-associated differences in genes related to lipid metabolism and adipocyte biology. *AGPAT2*, *CEBPB*, and *SREBF1* showed higher expression in YN pigs, whereas *FABP4* showed lower expression. These genes are associated with different aspects of lipid metabolism, including glycerolipid synthesis, transcriptional regulation of adipogenesis, and fatty acid handling [27,28]. Their expression patterns, considered alongside the higher IMF content observed in YN pigs, suggest that the two breeds may differ in the transcriptional regulation of muscle lipid metabolism. Nevertheless, the expression of individual genes does not directly establish changes in metabolic flux, lipid deposition, or fatty acid composition. In particular, the lower expression of *FABP4* indicates that not all genes associated with lipid metabolism changed in the same direction, highlighting the complexity of breed-associated transcriptional differences. Functional enrichment analysis of the definitive DEG set revealed that, among transcripts more highly expressed in L pigs, only the translation-related GO term remained significant after Benjamini–Hochberg correction (adjusted *p* = 7.6 × 10^−6^), whereas ribosome (adjusted *p* = 1.8 × 10^−3^) and oxidative phosphorylation (adjusted *p* = 1.3 × 10^−2^) were the only significant KEGG pathways. No GO term or KEGG pathway was significantly enriched among transcripts more highly expressed in YN pigs. The significant enrichment of translation-related GO terms and the ribosome pathway suggests that transcripts associated with the translational machinery, particularly those encoding ribosomal components, are more highly expressed in L muscle. Oxidative phosphorylation was also significantly enriched, indicating breed-associated differences in the expression of genes involved in mitochondrial energy metabolism. These findings may point to differences between breeds in the molecular processes underlying protein synthesis and mitochondrial energy metabolism. However, pathway enrichment reflects the overrepresentation of differentially expressed transcripts rather than direct measurements of biological activity. Therefore, these results do not establish differences in protein synthesis rates, translational activity, mitochondrial respiration, or energy-production capacity. Instead, they identify candidate biological processes potentially associated with the observed breed differences that warrant further functional investigation. Overall, the transcriptomic results identify candidate biological processes associated with breed differences rather than demonstrating the mechanisms responsible for the observed meat quality traits.

Lipidomic analysis provided complementary information on the composition of the muscle lipid pool. Among the differential lipid species, the relative contributions of individual molecular species varied across lipid classes. This pattern was particularly evident in the TG class, in which species containing an 18:1 or 20:1 fatty acyl chain accounted for 74.8% of the class-specific signal in YN pigs, compared with 45.3% in L pigs. These results indicate a difference in the distribution of TG molecular species between breeds. However, the proportions represent relative signal contributions within the TG class and should not be interpreted as direct measurements of absolute fatty acid concentrations or total lipid abundance. Intramuscular TG can serve as a reservoir of fatty acyl chains that may contribute to volatile formation through hydrolysis and subsequent oxidation during processing [29]. The fatty acyl-chain composition of muscle lipids may therefore be relevant to the potential formation of aroma compounds. Previous studies have also associated differences in unsaturated fatty acid profiles with variation in meat aroma characteristics [30,31]. In the present study, the lipidomic findings describe the composition of lipids in raw muscle rather than the rates or products of flavor-forming reactions during cooking. Although the observed lipid composition differences provide a plausible biochemical context for the breed-associated volatile profiles, the data do not demonstrate that the identified lipid species directly generated the differential volatile compounds. It is worth noting that the observed correlations among transcriptomic, lipidomic, and flavoromic features may primarily reflect breed-associated differences rather than independent relationships among these molecular features. After adjustment for breed and FDR correction, no volatile–gene or gene–lipid associations remained statistically significant. These findings highlight the need for further investigation to disentangle breed-associated effects from potential inter-omic relationships. Five transcripts, including *ATP5ME*, *DYNLL1*, *PFDN2*, *HIST1H2BD*, and *RPS29*, showed high connectivity in both correlation layers involving gene expression. These transcripts are associated with diverse cellular functions and are not, as a group, specific markers of lipid metabolism. Their network positions should therefore be interpreted as descriptive features of the observed cross-omics covariance rather than evidence that they directly regulate lipid composition or flavor formation. Furthermore, because the correlation analysis included samples from both breeds, some associations may reflect shared between-breed differences across multiple omics layers rather than direct relationships between individual molecular features. Accordingly, the network is best viewed as a hypothesis-generating representation of cross-omics associations. In contrast to these network-derived features, *CEBPB* and *SREBF1* were considered candidate genes based on their expression differences and established associations with lipid metabolism [32,33]. Their higher expression in YN pigs, together with the greater IMF content and distinct TG molecular-species profile observed in this breed, supports further investigation of their potential involvement in breed-associated differences in muscle lipid metabolism. However, the integration was based on only eight animals (four per breed) and involved a large number of pairwise correlations; therefore, the cross-omics associations should be interpreted as exploratory and hypothesis-generating. Moreover, the marked attenuation of correlations after adjustment for breed, together with the absence of gene-volatile and gene-lipid associations that remained significant after Benjamini–Hochberg correction, indicates that the unadjusted network structure was largely influenced by breed-associated differences. Accordingly, the tripartite map is presented as a descriptive representation of cross-omics co-variation rather than evidence of statistically validated biological relationships or regulatory mechanisms. Independent validation in larger cohorts is needed to assess whether these associations are reproducible and biologically meaningful. Nevertheless, the present study does not establish that either gene causes the observed lipidomic or volatile differences, nor does it demonstrate a direct regulatory pathway connecting these genes to specific aroma compounds. Functional validation and analyses of the relevant metabolic processes will be needed to determine whether these candidate genes contribute mechanistically to the observed phenotypes.

Collectively, this study identifies breed-associated differences in the raw-muscle volatile profile, lipid composition, and transcriptome of YN and L pigs. The integrated findings suggest potential relationships among transcriptional variation in lipid-related genes, the distribution of muscle lipid species, and volatile composition. From a theoretical perspective, this multi-omics framework provides a basis for generating and testing hypotheses about the molecular and metabolic factors associated with pork quality differences between pig breeds. From an applied perspective, the findings may inform further evaluation of the characteristic meat quality attributes of YN pigs. However, whether the observed chemical differences translate into perceptible sensory advantages, particularly after cooking, remains to be established through targeted analysis of heat-generated volatiles and appropriately designed sensory evaluation.

## 5. Conclusions

In summary, Yunong Black (YN) and Landrace (L) pigs exhibited distinct volatile profiles, muscle lipid compositions, and transcriptomic patterns. Relative odor activity value (ROAV) analysis highlighted several volatile compounds with potentially important contributions to odor characteristics, while transcriptomic and lipidomic analyses revealed breed-associated differences in lipid-related gene expression and muscle lipid composition. Although exploratory integrative correlation analysis identified strong pairwise correlations among breed-differential features, none of the volatile–gene or gene–lipid associations remained significant after adjustment for breed and Benjamini–Hochberg false discovery rate correction. These findings indicate that the observed correlations may primarily reflect breed-associated differences rather than independent relationships among molecular features. Overall, this study provides a multi-omics characterization of breed-associated variation in pork flavor-related traits and a basis for generating hypotheses for future investigations. Further studies are needed to clarify the molecular relationships underlying these differences and to validate the potential contributions of individual compounds to pork aroma through sensory evaluation.

## Figures and Tables

**Table 1:** Quality traits of YN and L pig muscle samples.

Items	L	YN	*p* Value
pH 45 min	6.06 ± 0.08 ^b^	6.20 ± 0.04 ^a^	0.005
pH 24 h	5.65 ± 0.01 ^b^	5.89 ± 0.03 ^a^	0.001
L*	53.39 ± 0.61 ^a^	49.70 ± 0.79 ^b^	<0.001
A*	6.49 ± 0.62 ^b^	11.20 ± 0.71 ^a^	<0.001
B*	7.71 ± 0.42	8.29 ± 0.63	0.123
IMF (g/100 g)	1.68 ± 0.41 ^b^	3.51 ± 0.36 ^a^	0.001

^a,b^ Means within a row with different subscripts differ when *p* value < 0.05. L, Landrace pig group; YN, Yunong black pig group.

**Table 2:** The 40 most significant annotated DEGs in the longissimus thoracis et lumborum muscle of YN and L pigs.

Gene ID	BaseMean	Fold Change (YN vs. L)	log_2_ Fold Change (YN vs. L)	Adjusted *p*-Value	Gene Name	Description	Regulation (YN vs. L)
ENSSSCG00000035417	103.59	9.56	3.26	2.67355 × 10^−13^	*TMEM238*	transmembrane protein 238	higher in YN
ENSSSCG00000029241	430.27	5.85	2.55	1.98258 × 10^−12^	*ASB10*	ankyrin repeat and SOCS box containing 10	higher in YN
ENSSSCG00000025190	1082.51	5.10	2.35	1.16806 × 10^−9^	*COQ10A*	coenzyme Q10A	higher in YN
ENSSSCG00000040089	185.87	7.86	2.97	1.16806 × 10^−9^	*TEDC1*	tubulin epsilon and delta complex 1	higher in YN
ENSSSCG00000017391	257.98	5.03	2.33	1.16806 × 10^−9^	*PLEKHH3*	pleckstrin homology, MyTH4 and FERM domain containing H3	higher in YN
ENSSSCG00000034820	249.27	5.07	2.34	1.30392 × 10^−9^	*MAFA*	MAF bZIP transcription factor A	higher in YN
ENSSSCG00000031280	650.81	5.27	2.40	3.96587 × 10^−9^	*MRPL57*	mitochondrial ribosomal protein L57	higher in YN
ENSSSCG00000038911	702.73	3.91	1.97	4.65654 × 10^−8^	*KLF2*	Kruppel-like factor 2	higher in YN
ENSSSCG00000008041	1783.97	3.75	1.91	7.65978 × 10^−8^	*PKD1*	polycystin 1, transient receptor potential channel interacting	higher in YN
ENSSSCG00000029944	741.64	5.81	2.54	1.56542 × 10^−7^	*FASN*	fatty acid synthase	higher in YN
ENSSSCG00000026457	297.44	3.98	1.99	1.56542 × 10^−7^	*GALNT17*	polypeptide N-acetylgalactosaminyltransferase 17	higher in YN
ENSSSCG00000029156	682.48	3.71	1.89	1.58616 × 10^−7^	*ZNF865*	zinc finger protein 865	higher in YN
ENSSSCG00000016364	4335.44	3.41	1.77	4.19341 × 10^−7^	*GPC1*	glypican 1	higher in YN
ENSSSCG00000003196	295.29	3.83	1.94	4.671 × 10^−7^	*PTOV1*	PTOV1 extended AT-hook containing adaptor protein	higher in YN
ENSSSCG00000005839	539.97	3.52	1.82	7.02546 × 10^−7^	*FBXW5*	F-box and WD repeat domain containing 5	higher in YN
ENSSSCG00000034773	2253.52	4.66	2.22	7.89808 × 10^−7^	*KCNJ18*	potassium inwardly rectifying channel subfamily J member 18	higher in YN
ENSSSCG00000017379	58.72	5.70	2.51	8.22567 × 10^−7^	*ETV4*	ETS variant transcription factor 4	higher in YN
ENSSSCG00000024665	321.96	3.56	1.83	1.80844 × 10^−6^	*TRAPPC12*	trafficking protein particle complex 12	higher in YN
ENSSSCG00000033456	4741.09	3.27	1.71	1.86151 × 10^−6^	*GPR157*	G protein-coupled receptor 157	higher in YN
ENSSSCG00000040025	217.48	3.73	1.90	2.0721 × 10^−6^	*NANOS1*	nanos C2HC-type zinc finger 1	higher in YN
ENSSSCG00000038507	1920.66	0.20	−2.30	4.89069 × 10^−11^	*RPS29*	ribosomal protein S29	higher in L
ENSSSCG00000021411	56.752	0.09	−3.49	8.10309 × 10^−11^	*HIST1H2BD*	histone cluster 1, H2bd	higher in L
ENSSSCG00000036724	5777.86	0.23	−2.13	6.91908 × 10^−10^	*CRYAB*	crystallin alpha B	higher in L
ENSSSCG00000006335	128.51	0.15	−2.72	3.84759 × 10^−9^	*RGS4*	regulator of G protein signaling 4	higher in L
ENSSSCG00000027072	119.13	0.12	−3.08	4.12111 × 10^−8^	*ATP5IF1*	ATP synthase inhibitory factor subunit 1	higher in L
ENSSSCG00000008842	72.22	0.16	−2.67	1.18219 × 10^−7^	*KIT*	KIT proto-oncogene, receptor tyrosine kinase	higher in L
ENSSSCG00000006308	454.66	0.25	−1.97	1.32932 × 10^−7^	*CREG1*	cellular repressor of E1A-stimulated genes 1	higher in L
ENSSSCG00000040681	2236.82	0.18	−2.46	1.43885 × 10^−7^	*FABP4*	fatty acid-binding protein 4, adipocyte	higher in L
ENSSSCG00000018086	1412.41	0.26	−1.95	1.56542 × 10^−7^	*ND4L*	NADH dehydrogenase subunit 4L	higher in L
ENSSSCG00000013297	210.78	0.24	−2.03	5.10669 × 10^−7^	*CD44*	CD44 molecule	higher in L
ENSSSCG00000009904	40.16	0.13	−2.92	8.91943 × 10^−7^	*DYNLL1*	dynein light-chain LC8-type 1	higher in L
ENSSSCG00000031128	91.36	0.21	−2.24	1.22418 × 10^−6^	*PPIC*	peptidylprolyl isomerase C	higher in L
ENSSSCG00000023903	571.01	0.28	−1.81	1.28868 × 10^−6^	*ATP5ME*	ATP synthase membrane subunit e	higher in L
ENSSSCG00000000579	163.98	0.25	−1.98	1.62257 × 10^−6^	*GOLT1B*	golgi transport 1B	higher in L
ENSSSCG00000040273	2737.72	0.31	−1.70	1.74894 × 10^−6^	*RPL35A*	ribosomal protein L35a	higher in L
ENSSSCG00000006366	220.70	0.27	−1.88	2.33345 × 10^−6^	*PFDN2*	prefoldin subunit 2	higher in L
ENSSSCG00000023374	218.47	0.25	−1.97	3.14231 × 10^−6^	*SRGN*	serglycin	higher in L
ENSSSCG00000009886	1029.94	0.28	−1.83	5.19479 × 10^−6^	*TRAFD1*	TRAF-type zinc finger domain containing 1	higher in L
ENSSSCG00000010292	926.33	0.32	−1.63	7.54368 × 10^−6^	*P4HA1*	prolyl 4-hydroxylase subunit alpha 1	higher in L
ENSSSCG00000033368	111.17	0.17	−2.55	7.84624 × 10^−6^	*ATP5MC2*	ATP synthase membrane subunit c locus 2	higher in L

## Data Availability

The raw RNA-seq reads have been deposited in NCBI SRA (BioProject accession PRJNA1536492), and the lipidomics and flavoromics raw and processed data have been deposited in MetaboLights (accession MTBLS15951). All datasets are accessible through the corresponding public repositories.

## Supplementary Materials

The following supporting information can be downloaded at: https://www.mdpi.com/article/10.3390/foods15213973/s1, Figure S1: Composition of the odour-activity profile of YN and L pork; Figure S2: Lipidomics normalisation diagnostics; Table S1: Composition and nutrient levels of the basal diet for pigs weighing 50–120 kg; Table S2: Curated lipidomics dataset with per feature statistics; Table S3: Differential expression statistics for the YN and L transcriptome comparison; Table S4: Identification and differential statistics of the volatile compounds; Table S5: Pairwise correlation statistics underlying the exploratory integrated multi-omics analysis; Table S6: Relative odour activity values, odour thresholds and odour descriptor summary; Table S7: Sensitivity of the lipidomics results to the choice of normalisation method; Table S8: Lipidomics Standards Initiative reporting checklist for the lipidomics analysis.
