# Supplementary material for: Volatile, Lipidomic and Transcriptomic Profiles of Longissimus Muscle from Yunong Black and Landrace Pigs: An Exploratory Comparison
Source: Foods. 2026 Nov 5;15(21):3973. doi: 10.3390/foods15213973 (PMC13763303; doi:10.3390/foods15213973)
Supplement: Supplementary file 1 [file foods-15-03973-s001.zip › Supplementary Figure S1 Composition of the odour-activity profile of YN and L pork.pdf]

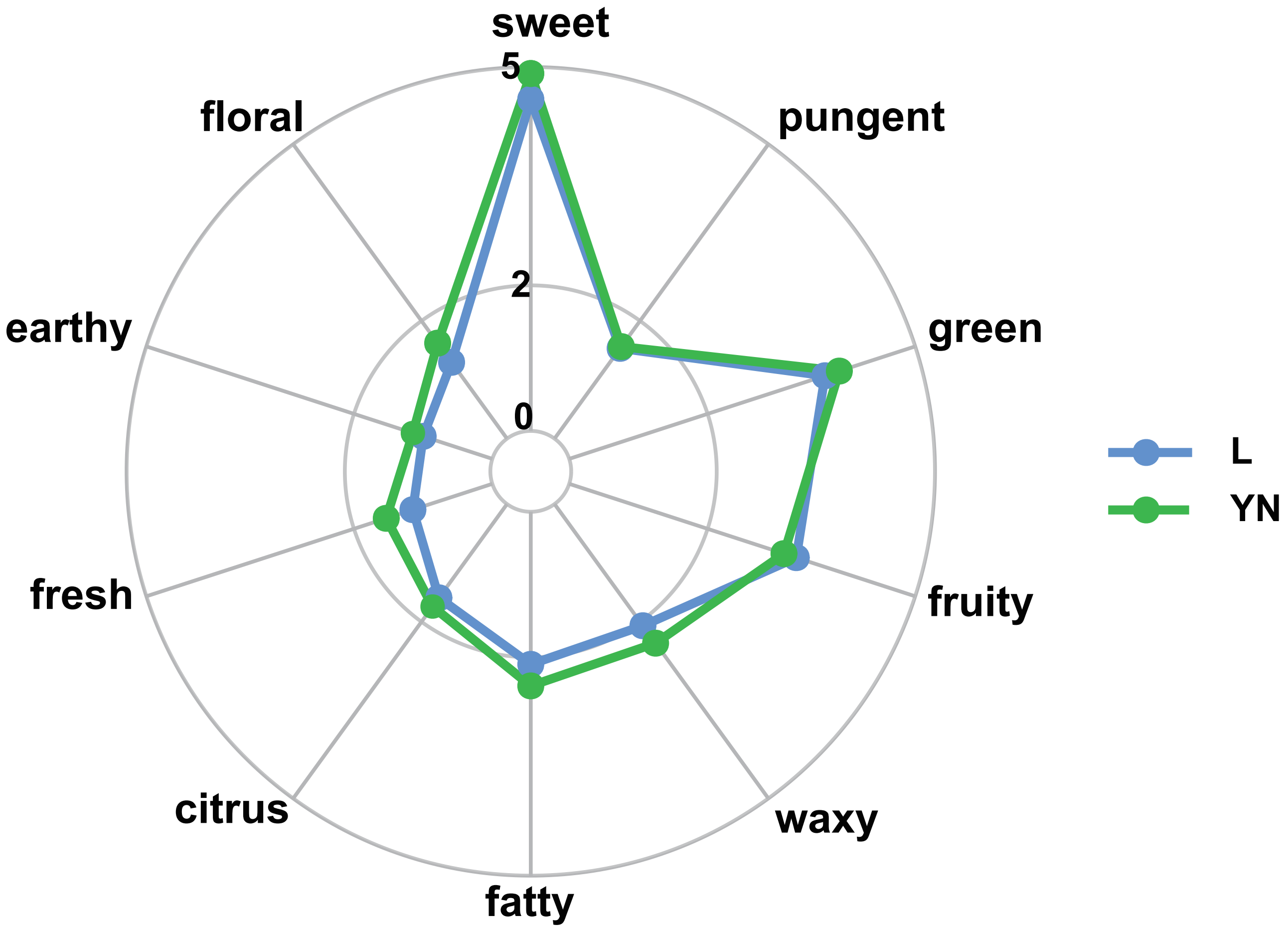

sweet
pungent
green
fruity
waxy
fatty
citrus
fresh
earthy
floral
5
2
0
L
YN
