# Supplementary material for: Volatile, Lipidomic and Transcriptomic Profiles of Longissimus Muscle from Yunong Black and Landrace Pigs: An Exploratory Comparison
Source: Foods. 2026 Nov 5;15(21):3973. doi: 10.3390/foods15213973 (PMC13763303; doi:10.3390/foods15213973)
Supplement: Supplementary file 1 [file foods-15-03973-s001.zip › Supplementary Figure S2 lipidomics normalisation diagnostics.pdf]

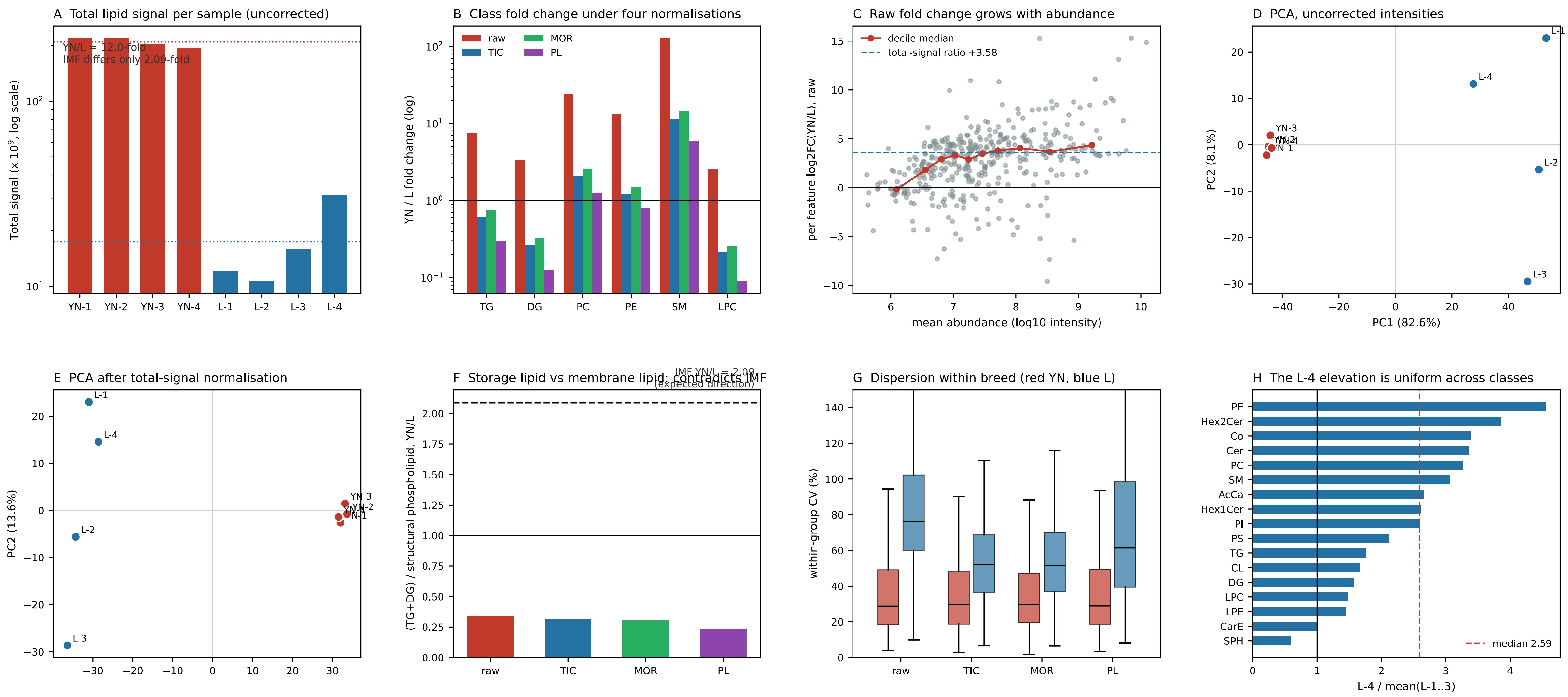
A Total lipid signal per sample (uncorrected)
YN/L = 12.0-fold
IMF differs only 2.09-fold
Total signal (x 10^9, log scale)
YN-1
YN-2
YN-3
YN-4
L-1
L-2
L-3
L-4
B Class fold change under four normalisations
raw
TIC
MOR
PL
YN / L fold change (log)
TG
DG
PC
PE
SM
LPC
C Raw fold change grows with abundance
decile median
total-signal ratio +3.58
per-feature log2FC(YN/L), raw
mean abundance (log10 intensity)
D PCA, uncorrected intensities
PC2 (8.1%)
PC1 (82.6%)
E PCA after total-signal normalisation
PC2 (13.6%)
F Storage lipid vs membrane lipid: contradicts IMF
IMF YN/L = 2.09
(expected direction)
(TG+DG) / structural phospholipid, YN/L
G Dispersion within breed (red YN, blue L)
within-group CV (%)
H The L-4 elevation is uniform across classes
PE
Hex2Cer
Co
Cer
PC
SM
AcCa
Hex1Cer
PI
PS
TG
CL
DG
LPC
LPE
CarE
SPH
median 2.59
L-4 / mean(L-1..3)
